# Exploring the relationship between interoception and mental body representation in Parkinson’s disease

**DOI:** 10.1007/s00702-026-03194-0

**Published:** 2026-06-06

**Authors:** Alfonsina D’Iorio, Chiara Alves, Francesca Pisano, Giovanni Luca Di Benedetto, Mariachiara Gaita, Carmine Vitale, Marianna Amboni, Simona Raimo, Liana Palermo, Gabriella Santangelo

**Affiliations:** 1 Department of Psychology, University of Campania “Luigi Vanvitelli”, Caserta, Italy; 2 Department of Medical, Motor and well-being Sciences, University Parthenope, Naples, Italy; 3 ICS Maugeri Hermitage, Naples, Italy; 4 Department of Medicine, Center for Neurodegenerative Diseases (CEMAND), University of Salerno, Salerno, Italy; 5 Department of Medical and Surgical Sciences, “Magna Graecia”, University of Catanzaro, Catanzaro, Italy

**Keywords:** Parkinson’s disease, Interoception, Mental body representation, Action-oriented body representation, Non-action-oriented body representation

## Abstract

Interoception refers to the process through which the nervous system detects, interprets, and integrates internal bodily signals, forming a dynamic representation of the body’s internal state at both conscious and unconscious levels. It is commonly conceptualized along three dimensions: interoceptive accuracy, interoceptive awareness and interoceptive sensitivity. Mental body representation (MBR) is a multifaceted cognitive construct encompassing structural, spatial, lexical, and semantic components of the body. A widely accepted taxonomy distinguishes between two types of MBR: action-oriented representations and non–action-oriented representations. To date, only a few studies have examined interoception and MBR in Parkinson’s disease (PD), often yielding inconsistent findings, and no study has directly investigated the relationship between these two domains in this clinical population. This study therefore aims to (1) further characterize interoceptive and MBR profiles in PD and (2) explore their potential association. Twenty-seven patients with PD and twenty-four healthy controls completed a neuropsychological battery assessing global cognitive functioning, interoceptive abilities, MBR, and depressive and apathetic symptoms. Mann–Whitney analyses revealed significantly poorer MBR performance in patients compared with healthy controls. Spearman correlations showed significant associations between action-oriented MBR and global cognitive functioning as measured by the Parkinson’s Disease Cognitive Rating Scale (PD-CRS). Regression analyses further demonstrated that PD-CRS performance significantly predicted action-oriented MBR abilities. These findings indicate a characteristic difficulty among individuals with PD in mentally representing body features. Moreover, results suggest that both cortical and subcortical cognitive functions contribute to supporting action-oriented MBR.

**Supplementary Information:**

The online version contains supplementary material available at https://doi.org/10.1007/s00702-026-03194-0.

## Introduction

Parkinson’s disease (PD) is the most common movement disorder, characterized by both motor and non-motor symptoms (Aarsland et al. 2021).

Despite the extensive literature on PD, interoception and mental body representation remain two underexplored non-motor symptoms in this condition, with no studies investigating their potential relationship.

Interoception is the process by which the nervous systems sense, integrate, and interpret signals originating from within the body, providing a representation of the organism’s internal state (Berntson and Khalsa 2021). Its central processing relies on a hierarchical network involving subcortical and cortical regions, with a pivotal role played by the insular cortex in integrating interoceptive, emotional, and cognitive information (Chen et al. 2021). Interoception can be operationalized along three distinct dimensions: (a) *interoceptive accuracy* (IAc), the objective precision and reliability to detect internal bodily signals; (b) *interoceptive awareness* (IAw), the metacognitive insight into interoceptive accuracy; and (c) *interoceptive sensitivity* (IS), the subjective tendency to perceive internal bodily signals (Garfinkel et al. 2015). Alterations of interoception have been reported in several psychiatric and neurological conditions, including schizophrenia and Alzheimer’s disease (Khalsa et al. 2018). Such interoceptive alterations have scarcely been documented in PD, where studies suggest impairments in both IAc and IS (Ricciardi et al. 2016; Santangelo et al. 2018), with worse impairments in individuals with the tremor-dominant motor subtype compared with those presenting the rigidity–bradykinesia phenotype (Santangelo et al. 2018).

Mental body representation (MBR) is a cognitive domain encompassing structural, spatial, semantic, and lexical body knowledge (Grossi and Trojano 2011). Its central processing relies on a distributed bilateral network involving frontal, parietal, temporal, and occipital regions, with partially dissociable substrates for specific form of representation as well as shared midline structures, including the precuneus and medial frontal regions (Di Vita et al. 2016a). MBR can be operationalized along two different forms: a *dynamic*,* action-oriented representation* (body schema), supporting planning, execution, and monitoring of movements, and a *static*,* non-action-oriented representation* (body image), include body-part recognition, spatial body knowledge, and self-related processing (De Vignemont 2010; Di Vita et al. 2016b). Distortions of MBR are a core feature of several psychiatric and neurological conditions, including schizophrenia and frontotemporal dementia (Longo 2022). Such MBR alterations have scarcely yet conflictively been documented in PD, with some studies suggesting a total impairment of this ability (Dominey et al. 1995; Helmich et al. 2007; Bek et al. 2022), and others indicating a partial (Scarpina et al. 2019) or a total (Van Nuenen et al. 2012) preservation of this cognitive function.

Since interoception and MBR have so far received limited attention in the context of PD and given their relevance for everyday functioning, the present study aims to assess both domains in patients with PD (PwPD). Indeed, we should take into account that interoception underlies a range of functional abilities, including energy regulation, affective experience, self-consciousness, decision-making and social cognition (Brewer et al. 2021). Moreover, recent studies suggest dysfunction of critical interoceptive hubs in PD, such as the insular cortex (Santangelo et al. 2018); thus, it is necessary to further investigate PD impact on interoception. On the other side, MBR underlies a range of functional abilities, including bodily self-consciousness, motor coordination, and sensorimotor integration (Möllmann et al. 2024). Specifically, recent studies highlight that impairments in body schema tend to affect motor planning and execution, potentially contributing to motor deficits (Abraham et al. 2021); this evidence underscores the clinical relevance of investigating how a motor disorder such as PD may affect MBR abilities.

A further purpose was to examine for the first time the relationship between interoception and MBR in PD, as current evidence indicates that interoceptive processing contributes to both body schema construction and body image formation (Raimo et al. 2021a). Investigating this relationship may also have important clinical implications, as establishing a link between interoception and MBR in PD could inform the development of targeted interventions aimed at improving self-awareness, motor planning, and body representation deficits.

Our hypotheses were: (1) that PwPD may exhibit an altered interoceptive ability, (2) that PwPD may exhibit an altered MBR ability, (3) that interoception and MBR would be correlated. No more hypotheses were formulated, as the other cognitive tasks included, as explained below, have been used as screening tools to exclude patients with possible cognitive decline that could have confounded task performance and, consequently, the interpretation of the experimental results.

## Methods

### Participants

The present study included a total of 51 participants, comprising 27 PwPD (14 males, 13 females) and 24 healthy control subjects (HCs; 8 males, 16 females).

Patients were recruited as outpatients from IDC-Hermitage Capodimonte in Naples, Italy, according to the following inclusion criteria: (1) clinical diagnosis of idiopathic PD according to the diagnostic guidelines proposed by Gelb et al. (Gelb et al. 1999); (2) preserved global cognitive functioning, defined by a score > 15.5 on the Montreal Cognitive Assessment (MoCA) (Santangelo et al. 2015); and (3) absence of any additional neurological/psychiatric diseases.

HCs were selected according to the following inclusion criteria: (1) 40–80 years old; (2) preserved global cognitive functioning, indicated by a score > 15.5 on the MoCA (Santangelo et al. 2015); and (3) absence of previous or actual diagnoses of neurological/psychiatric diseases.

Demographic data (i.e., sex, age, years of education) and clinical characteristics — including disease duration, severity of motor symptoms, assessed by the Unified Parkinson’s Disease Rating Scale - part III (UPDRS-III) (Fahn 1987), and disease staging, measured by the Hoehn and Yahr scale (H&Y) (Hoehn and Yahr 1967)— were recorded.

All patients were assessed in “ON” medication state.

All participants provided written informed consent prior to inclusion. The study was conducted according to the guidelines of the Declaration of Helsinki (1964).

### Measures

Participants underwent a neuropsychological battery to assess global cognitive functioning, interoception, MBR and the occurrence of both depressive and apathetic symptoms.

Global cognitive functioning was assessed using both the MoCA (Santangelo et al. 2015) and the Parkinson’s Disease Cognitive Rating Scale (PD-CRS/AF) (D’Iorio et al. 2022).

Specifically, the MoCA is a brief cognitive screening tool composed of 30 items evaluating eight cognitive domains: short- and long-term verbal memory, visuospatial abilities, executive functions, attention, concentration, working memory, language, and spatial and temporal orientation. The total raw score ranges from 0 (worst performance) to 30 (best performance) and is corrected for age and education. Based on the Italian validation, a corrected score ≤ 15.5 indicates a cognitive impairment (Santangelo et al. 2015).

Similarly, the PD-CRS/AF is a PD-specific cognitive screening scale composed of 9 items assessing both cortical (naming, sustained attention, working memory, clock drawing and copy drawing) and fronto-subcortical functions (immediate memory, delayed memory, alternating fluency, and action fluency). The total raw score ranges from 0 (worst performance) to 134 (best performance) and is corrected for age and education. According to the Italian validation, a corrected score ≤ 62.89 indicates a cognitive impairment (D’Iorio et al. 2022).

Interoceptive ability was assessed using the Heartbeat Perception Task (HbPT), for IAc and IAw (Schandry 1981), and the Self-Awareness Questionnaire (SAQ), for IS (Longarzo et al. 2015).

In the HbPT, participants silently count their heartbeats during time intervals of predetermined but undisclosed duration, without relying on external cues (Schandry 1981). The task includes two trials, each comprising three counting intervals (35 s, 25 s, and 45 s), presented in this fixed order and separated by 20-second pauses. The beginning and end of each interval are indicated by auditory signals (“start” and “stop,” respectively). After each interval, participants report the number of heartbeats counted and rate their confidence in the accuracy of their count on a scale ranging from 0 (“not at all confident”) to 10 (“very confident”). Throughout both trials, participants wear a Polar Sense wrist device, which records the actual number of heartbeats, providing the IAc measure. For each of the six intervals, IAc is calculated as: 1– (|n real beats – n counted beats|)/((n real beats + n counted beats)/2); a mean of these six scores provides a single IAc value for each participant, with higher values indicating greater accuracy. Subsequently, IAw is assessed by the correlation between the global IAc score and the confidence ratings in estimating heartbeats during the HbPT. At the end of the two trials, participants are describe how they performed the task while counting their heartbeats and indicate, on a structural body map, the body area where they perceived their heartbeats; if no heartbeat is perceived, they mark an “X” in the box labeled “nowhere” above the body map (adapted from Plans et al. (Plans et al. 2021).

The SAQ includes 35 items concerning the frequency with which individuals perceive visceral and somatosensory bodily sensations. These sensations involve different bodily domains, including cardiac (e.g., feeling the heartbeat pulsing in the ears or experiencing palpitations), respiratory (e.g., shortness of breath or chest tightness), gastrointestinal (e.g., stomach tightness, nausea, bloating, hunger, thirst, or urgency to urinate/defecate), thermoregulatory (e.g., feeling excessively hot or cold compared to others), somatic (e.g., back pain, tingling, itching, heavy legs, shifting pain), and throat-related sensations (e.g., dry throat, lump in the throat, difficulty swallowing); the questionnaire also includes items related to autonomic activation, such as sweaty hands, dizziness, or faint feeling. Responses are given on a 5-point Likert scale (0 = never, 1 = sometimes, 2 = often, 3 = very often, 4 = always). The total raw score ranges from 0 to 140, with higher scores indicating heightened attention to internal bodily sensations (Longarzo et al. 2015).

The MBR was assessed using a computer-based test battery developed by Raimo et al. (Raimo et al. 2021c) (see also Raimo et al. (Raimo et al. 2025), comprising two primary tasks: the Frontal Body Evocation task (FBE; modified from Daurat-Hmeljiak et al. (Daurat-Hmeljiak et al. 1978), and the Hand Laterality Task (HLT; adapted and simplified from Parsons (Parsons 1987) and Fontes et al. (Fontes et al. 2014). The battery also includes two control tasks, allowing to evaluate the MBR independently of task-specific aspects (i.e., visual processing, mental imagery, visuo-spatial attention, decision making): the Christmas Tree Task (CTT) (Raimo et al. 2021c) and the Object Laterality Task (OLT; modified from Piccardi et al. (Piccardi et al. 2011).

In the FBE task, assessing non–action-oriented MBR, participants are presented with a body image for approximately 10s; subsequently, nine body parts -left/right leg, hand, arm, chest side, and neck- appear sequentially on the right side of the frame and must be dragged to the correct position using the head as reference. Accuracy is the total deviation in millimeters between the placed and correct positions, with higher scores indicating poorer performance; four different accuracy scores are collected: right-side, left-side, central -neck-, and total score.

In the HLT, assessing action-oriented MBR, participants are presented with a series of 20 dorsal-view hand images − 10 right, 10 left-, shown one at a time, each rotated from 0° to 90° clockwise or counterclockwise; below each image, a pair of comparison hands -right and left- is simultaneously displayed and participants select the one matching the target’s laterality. Accuracy is the total number of correct responses and ranges from 0 to 20, with higher scores indicating better performance; three different accuracy scores are obtained: right-side, left-side, and total score. This mechanism also applies to the corresponding control task (OLT), assessing mental rotation of non-body stimuli, where participants judge the laterality of flower according to their leaf side.

In the CTT, assessing the visuospatial processing of non-body stimuli, participants are presented with an image of a Christmas tree for approximately 10s; subsequently, nine tree parts -upper left/right branches, middle left/right branches, lower left/right branches, left/right pot section, and the top tip- appear sequentially on the right side of the frame and must be dragged to the correct position using the treetop as a reference. Accuracy is the total deviation in millimeters between the placed and correct positions, with higher scores indicating poorer performance.

In the OLT, assessing mental rotation of non-body stimuli, participants are presented with 20 flower images − 10 right-leafed, 10 left-leafed- shown one at a time, each rotated from 0° to 90° clockwise or counterclockwise; below each image, a pair of comparison flowers -right-leafed and left-leafed- is simultaneously displayed and participants select the one matching the target’s laterality. Accuracy is the total number of correct responses and ranges from 0 to 20, with higher scores indicating better performance.

Depression was assessed using the Beck Depression Inventory–II (BDI-II) (Maggi et al. 2023), a self-report questionnaire composed of 21 items assessing the severity of depressive symptoms over the past two weeks. The total score ranges from 0 to 63, with higher scores indicating greater depressive symptomatology; specifically, according to the Italian version for PD, a score ≥ 15.5 indicates a depressive symptomatology (Maggi et al. 2023).

Apathy was assessed using the Dimensional Apathy Scale (I-DAS) (Santangelo et al. 2017), a self-report questionnaire composed of 24 items assessing the frequency of apathetic symptoms over the past month. It considers apathy as a multidimensional construct, evaluating the three subtypes traditionally described in the literature: cognitive apathy, emotional–affective apathy and auto-activation deficit apathy. The total score ranges from 0 to 72, with higher scores indicating greater apathy severity; specifically, according to the Italian version for PD, a total score ≥ 28.5 indicates an apathetic symptomatology (Santangelo et al. 2017).

### Analysis

Mann–Whitney test was performed to compare PwPD and HCs’performances with respect to cognitive tasks and behavioural measures (MoCA, PD-CRS/AF, HbPT, SAQ, FBE, HLT, CTT, OLT, BDI-II and I-DAS). The Benjamini–Hochberg procedure was applied to adjust for multiple comparisons.

In addition, four non-parametric bivariate correlation analyses (Spearman’s rho) were conducted within the patients’ group. The first three analyses explored correlations of interoception and MBR with: (1) clinical variables (disease duration, severity of motor symptoms, and disease staging), (2) cognitive variables (MoCA and PD-CRS/AF), and (3) behavioural variables (depression and apathy). The fourth analysis explored the relationship between interoception (HbPT and SAQ) and MBR (FBE and HLT). The Benjamini–Hochberg procedure was applied to adjust for multiple comparisons in all correlation analyses.

Finally, stepwise regression analyses were performed exclusively on the variables that had shown significant associations in the previous correlation analyses, with the aim of identifying potential predictors of MBR ability.

All statistical analyses were conducted using Jamovi 2.6.44.0, adopting a significance threshold of α < 0.05.

## Results

Descriptive statistics were computed for clinical, demographic, cognitive, and behavioural variables.

Regarding demographic variables, no statistically significant differences emerged between the two groups in age (*U* = 285, *p* = 0.461, *ES*=-0.121), sex (*U* = 264, *p* = 0.191, *ES* = 0.185) or years of education (*U* = 228, *p* = 0.064, *ES*=-0.296).

Concerning tasks assessing interoception, no statistically significant differences were highlighted between the two groups for any of the three interoceptive dimensions (HbPT TOT-accuracy: *U* = 307, *p* = 0.755, *ES* = 0.052; HbPT TOT-awareness: *U* = 323, *p* = 0.992, *ES* = 0.003; SAQ: *U* = 238, *p* = 0.215, *ES* = 0.208).

Concerning tasks assessing MBR, statistically significant differences were observed between the two groups in the FBE task for the right body side (*U* = 187, *p* = 0.010, *ES*=-0.423) and in the HLT task for the right hands (*U* = 215, *p* = 0.004, *ES* = 0.338), as well as for total accuracy in the HLT task (*U* = 186, *p* = 0.004, *ES* = 0.427), with patients performing worse than HCs in all these tasks (FBE right body side score: *M* = 41.11, *SD* = 30.46 vs. *M* = 26.92, *SD* = 9.71, respectively; HLT right side score: *M* = 9.00, *SD* = 1.75 vs. *M* = 9.96, *SD* = 0.20, respectively; HLT total score *M* = 17.59, *SD* = 3.38 vs. *M* = 19.58, *SD* = 0.83, respectively).

Regarding behavioural variables, statistically significant differences emerged between the two groups in the emotional component of the I-DAS (*U* = 149, *p* < 0.001, *ES*=-0.540), with patients showing greater severity of apathy symptoms with respect to HCs (*M* = 9.22, *SD* = 3.52 vs. *M* = 6.04, *SD* = 2.91, respectively).

Full descriptive analyses results are provided in Table 1.

**Table 1:** Descriptive statistics and Mann-Whitney U test for demographic, clinical, cognitive, and behavioral variables in the two groups

	Patients (*n* = 27)	Healthy controls (*n* = 24)	U	*p*	ES
Demographic variables					
Age	61.11 ± 10.27; 61 (42 to 78)	59.50 ± 8.87; 58 (47 to 78)	285.000	0.461	– 0.122
Education	13 ± 4.17; 13 (3 to 18)	11.00 ± 3.40; 12 (5 to 18)	228.000	0.064	– 0.296
Sex (F/M)	13/14	16/8	264.000	0.191	0.185
Clinical variables					
Disease duration	9.52 ± 4.53; 10 (2 to 16)	–	–	–	–
UPDRS-III score	15.5 ± 9.52; 13 (3 to 36)	–	–	–	–
H&Y score	2.19 ± 0.54; 2 (1 to 3)	–	–	–	–
Cognitive variables					
MoCA score	22.37 ± 3.55; 22 (14 to 28)	23.25 ± 3.04; 23.5 (16 to 28)	277.000	0.372	0.147
PD-CRS/AF score	83.37 ± 18.10; 87 (48 to 116)	91.96 ± 11.50; 90.5 (73 to 119)	244.000	0.131	0.248
HbPT- accuracy score	0.05 ± 0.53; 0.20 (− 1 to 0.90)	0.17 ± 0.23; 0.16 (− 0.21 to 0.53)	307.000	0.755	0.052
HbPT- awareness score	0.33 ± 0.43; 0.28 (− 54 to 1)	0.32 ± 0.36; 0.36 (0.33 to 0.90)	323.000	0.992	0.003
SAQ score	23.72 ± 12.39; 20 (4 to 46)	27.83 ± 11.15; 29 (7 to 45)	238.000	0.215	0.208
FBE total score	87.24 ± 39.10; 79.6 (42.6 to 215)	68.85 ± 25.10; 60.8 (32.4 to 107)	228.000	0.072	– 0.296
FBE left side score	42.34 ± 18.93; 38.1 (17.1 to 85.3)	39.05 ± 17.88; 35.8 (15.3 to 76)	292.000	0.552	– 0.099
FBE central region score	3.78 ± 1.81; 3.27 (1.30 to 9.27)	2.89 ± 1.88; 2.71 (0.31 to 9.14)	222.000	0.054	– 0.316
FBE right side score	41.11 ± 30.46; 32.4 (18.4 to 176)	26.92 ± 9.71; 25.6 (10.3 to 49.4)	187.000	0.010*	– 0.423
HLT total score	17.59 ± 3.38; 19 (8 to 20)	19.58 ± 0.83; 20 (17 to 20)	186.000	0.004*	0.427
HLT left side score	8.59 ± 1.91; 9 (4 to 10)	9.63 ± 0.71; 10 (8 to 10)	207.000	0.014	0.361
HLT right side score	9.00 ± 1.75; 10 (4 to 10)	9.96 ± 0.20; 10 (9 to 10)	215.000	0.004*	0.338
CTT score	158.78 ± 75.48; 148 (56.4 to 376)	135.36 ± 38.02; 122 (73.1 to 241)	284.000	0.459	– 0.123
OLT score	18.22 ± 3.98; 20 (3 to 20)	19.71 ± 0.86; 20 (16 to 20)	239.000	0.047	0.262
Behavioural variables					
BDI-II score	9.81 ± 7.32; 7 (1 to 31)	7.63 ± 7.30; 5.5 (0 to 24)	256.000	0.199	− 0.211
I-DAS executive score	6.11 ± 4.31; 5 (0 to 17)	6.58 ± 4.33; 6.5 (0 to 14)	300.000	0.656	0.074
I-DAS emotional score	9.22 ± 3.52; 10 (0 to 15)	6.04 ± 2.911; 5.5 (2 to 12)	149.000	< 0.001*	− 0.540
I-DAS behavioural/cognitive score	8.19 ± 4.93; 8 (0 to 20)	6.46 ± 3.59; 6 (1 to 12)	256.000	0.201	− 0.210

Values are expressed as mean ± standard deviation; median (range)

*U* U-value Mann Whitney test, *ES* effect size, *F* female participants, *M* male participants, *UPDRS-III* Unified Parkinson’s disease rating scale - part III, *H&Y* Hoehn and Yahr scale, *MoCA* Montreal cognitive assessment, *PD-CRS/AF* Parkinson’s disease-cognitive rating scale/alternative form, *HbPT*: heartbeat perception task, *SAQ* self-awareness questionnaire, *FBE* frontal body evocation task, *HLT* hand laterality task, *CTT* Christmas tree task, *OLT* object laterality task, *BDI-II* beck depression inventory-II, *I-DAS* Italian-dimensional apathy scale

*Significant after Benjamini-Hochberg correction for multiple comparisons

With respect to the correlation analyses, no statistically significant associations emerged, except for significant positive correlations between accuracy scores on the HLT scores and PD-CRS/AF scores, indicating that higher HLT scores—reflecting a better performance—are associated with higher PD-CRS/AF scores — reflecting a better global cognitive functioning. More specifically, significant correlations emerged between PD-CRS/AF and HLT total score (*ρ* = 0.641, *p* < 0.001), between PD-CRS/AF and HLT left side score (*ρ* = 0.602, *p* < 0.001) and between PD-CRS/AF and HLT right side score (*ρ* = 0.518, *p* = 0.006).

Significant correlation analyses results are provided in Table 2.

**Table 2:** Correlational analyses, with Spearman’s rho coefficient, between PDCRS/AF and HLT in the patients’ group

	PDCRS/AF score
HLT left side score	rho = 0.602 *p* < 0.001*
HLT right side score	rho = 0.518 *p* = 0.006*
HLT total score	rho = 0.641 *p* < 0.001*

*PD-CRS/AF* Parkinson’s disease-cognitive rating scale/alternative form, *HLT* hand laterality task

*Significant after Benjamini-Hochberg correction for multiple comparisons

Full correlation analyses results are provided in Supplementary materials (Tables S1, S2, S3 and S4).

As regards regression analyses, results revealed PD-CRS/AF to be a significant predictor of all three subscores of action-oriented MBR., i.e., HLT total score (*R²* = 0.30, adjusted *R²* = 0.27, *F*(1,25) = 10.5, *β =* 0.10, *p* = 0.003), HLT right side score (*R²* = 0.20, adjusted *R²* = 0.17, *F*(1,25) = 6.37, *β* = 0.04, *p* = 0.002) and HLT left side score (*R²* = 0.30, adjusted *R²* =0.27, *F*(1,25) = 10.7, *β =* 0.06, *p* = 0.003).

## Discussion

The present study sought to examine both interoceptive and body representation abilities, as well as their potential association, in PwPD. We hypothesized that PwPD may exhibit an altered interoceptive ability, according to the present literature highlighting alterations in interoceptive sensitivity and accuracy in PD (Ricciardi et al. 2016; Santangelo et al. 2018). We also hypothesized that PwPD may exhibit an altered ability in MBR, in line with recent literature suggesting dysfunctions, in PD, of cerebral regions—such as the primary motor cortex and the primary somatosensory cortex—which are shown to be critical for processing both action-oriented and non-action-oriented MBR (Di Vita et al. 2016b). As for the potential association between interoception and MBR, we expected a significant correlation between them, as current evidence supports the view that interoceptive processing plays a key role both in the construction of the body schema and in the formation of the body image (Raimo et al. 2021a).

Results revealed no significant differences between patients and HCs across the three interoceptive dimensions assessed. This result contrasts with previous reports documenting interoceptive alterations, as reported below (Ricciardi et al. 2016; Santangelo et al. 2018). Several explanations may account for this discrepancy, including methodological differences. For instance, Ricciardi et al. (Ricciardi et al. 2016) assessed interoceptive sensitivity (which corresponds to our construct of IAcc) using different parameters in the HbPT, including the number of trials (three in their study vs. six in ours), the inter-trial rest interval (30 s in their protocol vs. 20 s in ours), and the accuracy scoring formula (their 1/3 ∑ [1 − (|recorded heartbeats − counted heartbeats| / recorded heartbeats)] vs. our 1 − (|n real beats − n counted beats|) / ((n real beats + n counted beats)/2). On the other hand, Santangelo et al. (Santangelo et al. 2018) reported an association between interoception and motor subtype, specifically showing a reduction of both interoceptive sensibility and accuracy in the tremor-dominant phenotype compared to the rigidity–bradykinesia phenotype (Santangelo et al. 2018). Therefore, this discrepancy may be explained by the fact that, in the present study, the sample was not subdivided according to motor subtype.

As regards MBR abilities, significant group differences were observed in tasks assessing static and dynamic body representations (i.e., FBE and HLT respectively), where PD patients performed worse than HCs. The absence of differences on the corresponding control tasks (CTT and OLT) indicates that these impairments cannot be attributed to general linguistic, perceptual, attentional demands; instead, the findings point to a domain-specific difficulty in mentally representing sensorimotor aspects, structural, and visuo-spatial, features of the body. Importantly, no significant between-group differences were found in demographic variables (age, sex and education) or in global cognitive measures (MoCA and PD-CRS/AF). This strengthens the interpretation that the observed deficits in body representation are not secondary to demographic or cognitive confounders but instead reflect a specific alteration of MBR in PwPD.

Also, the present findings align closely with those reported by Dominey et al. (Dominey et al. 1995), Helmich et al. (Helmich et al. 2007), and Bek et al. (Bek et al. 2022), who likewise observed significant differences between PwPD and HCs on the HLT, typically reflected in slower responses and/or reduced accuracy in the patients’ group. Our results partially diverge from those of Scarpina et al. (Scarpina et al. 2019), who identified only a mild impairment in the more explicit component of the action-oriented body representation in PD, specifically when participants were required either to execute or imagine executing movements with their limbs (mental motor chronometry task), but not when using an hand laterality task. They also differ from the findings of van Nuenen et al. (Van Nuenen et al. 2012), who reported completely preserved MBR in PD. Several factors may account for these inconsistencies, related to methodological discrepancies, including sample characteristics, type of MBR tasks, “on” versus “off” medication state. First of all, many of the aforementioned studies recruited very small samples (e.g., 7 in Dominey et al. (Dominey et al. 1995); 12 in Helmich et al. (Helmich et al. 2007); 11 in van Nuenen et al. (Van Nuenen et al. 2012). Moreover, some studies included participants who markedly differed in age (e.g., Mean = 52 yrs. in Helmich et al. (Helmich et al. 2007) versus Mean = 65 yrs. in Bek et al. (Bek et al. 2022). In addition, certain studies stratified participants according to symptom lateralization (Scarpina et al. 2019) or included only right-lateralized patients (Helmich et al. 2007). Furthermore, these studies employed different versions of the HLT, and in some cases no control task was used at all (e.g. (Helmich et al. 2007). Also, all of these investigations examined MBR within the broader context of motor imagery, using the HLT as an implicit index of motor imagery; to date, no research has assessed MBR from a representational standpoint, incorporating a comprehensive evaluation of both action-oriented and non–action-oriented body representations within the same participant sample and using both MBR-specific and control tasks. Finally, these studies considered different medication states (e.g. in Dominey et al. (Dominey et al. 1995) five of seven patients were in “on” state; in both Helmich et al. (Helmich et al. 2007) and Van Nuenen (Van Nuenen et al. 2012) all patients were in “off” state; in both Scarpina et al. (Scarpina et al. 2019) and Bek et al. (Bek et al. 2022) all patients were in “on” state); this aspect further limits the direct comparability of findings, as dopaminergic treatment and motor state are known to differentially influence cognitive functioning in PD (Poletti and Bonuccelli 2013). However, we should consider that direct comparisons among these studies are inherently difficult—and, to some extent, methodologically inappropriate—because they vary greatly with respect to participant characteristics, study design, the specific tasks employed to assess MBR, and the clinical medication motor state.

Overall, these findings related to interoception and MBR may be explained by the fact that the sample recruited in the present study consists of patients in the early/intermediate stages of the disease (Mean = 9.52 years; SD = 4.53 years) and that alterations in critical interoceptive hubs, such as limbic and insular regions, are typically observed in the later stages of the disease (Braak et al. 2003), alongside cognitive impairment (Fathy et al. 2020). At the same time, it is plausible that deficits in the MBR are already evident from earlier stages of disease progression; in line with this hypothesis, current evidence agrees in indicating that the primary motor cortex, which plays a crucial role in processing action-oriented MBR, is already altered in the early phases of PD, due to dopaminergic depletion in the mesencephalon, from which motor cortex receives both direct and indirect inputs (Gaspar et al. 1991; Moore et al. 2008; Underwood and Parr-Brownlie 2021). Additionally, reduced functional connectivity between the precuneus and motor regions has been demonstrated in early stages PD patients (Thibes et al. 2017); these sites constitute important nodes for the processing of both action-oriented and non-action-oriented mental body representations (Corradi-Dell’Acqua et al. 2009; Di Vita et al. 2016c).

It is worth noting that the present study revealed a degree of asymmetry in body representation deficits, which appear to be more pronounced for the right side of the body in both tasks (FBE and HLT). Given the absence of differences on the corresponding control tasks (CTT and OLT), these impairments cannot be attributed to task difficulty and, thus, to a more general decline in mental rotation or visuospatial processing (Raimo et al. 2021b). Rather, they could be interpreted as reflecting the motor symptoms lateralization typically observed in PD; although no clinical data on motor lateralization were collected for the recruited patients, preventing a direct test of this interpretation, the current literature on this theme suggests that the mental representation of movement preserves a trace of clinical motor lateralization in PD (e.g., (Dominey et al. 1995; Helmich et al. 2007; Lambert et al. 2024). Specifically, Dominey et al. (Dominey et al. 1995) observed that patients with right-sided motor lateralization showed slower performance in manual finger sequencing task when using the right (affected) hand compared to the left (unaffected) hand; also, they were slower in identifying the affected right hand compared to the left hand in hand rotation tasks. Comparable results were reported by Helmich et al. (Helmich et al. 2007), who employed a classic hand laterality judgment task in parkinsonian patients characterized by marked right-sided motor lateralization, finding greater slowness in judging the laterality of the affected hand compared to the unaffected one. More recent evidence comes from Lambert et al. (Lambert et al. 2024), who investigated temporal accuracy of motor imagery in PwPD, finding a difference between the more versus less affected side, with significantly shorter mental versus physical movement times for the more affected side in a mental chronometry task. Taken together, these results suggest that mental representation retains the lateralization of motor deficits in PwPD, suggesting the possibility to interpret our results as reflecting the motor symptoms lateralization typically observed in PD; however, we did not collect data on motor symptoms lateralization, not allowing us to directly test this possibility.

Correlation analyses revealed significant positive correlations between accuracy scores on the HLT (i.e., HLT total score, HLT left side score and HLT right side score) and PD-CRS/AF scores, indicating that a better performance on the HLT, assessing action-oriented MBR, is associated to a better global cognitive functioning. In addition, regression analyses found PD-CRS/AF to be a significant predictor of HLT scores. Altogether, present results suggest that typical cognitive functions explored by PD-CRS/AF, both subcortical and cortical ones, rather than one assessed by MoCA, are propaedeutical to a proper and functional action-oriented representation of the body. This interpretation aligns with recent evidence indicating a shared neural substrate between action-oriented MBR and PD-CRS abilities. Specifically, Di Vita et al. (Di Vita et al. 2016b) demonstrated that a network including the precuneus and the medial frontal gyrus plays a crucial role in supporting action-oriented MBR. Notably, the same network appears to underlie both cortical and subcortical functions assessed by the PD-CRS (Brandão et al. 2024).

As for the potential association between interoception and MBR, the present results do not fit with current evidence, supporting a strict relationship between them (Coello and Fischer 2016; Badoud and Tsakiris 2017). A possible explanation for this result might be related to the absence of significant differences between PwPD and HCs in interoceptive tasks; it is possible that the limited variability in interceptive scores has reduced the likelihood of detecting correlations, even when a theoretical relationship between the two dimensions exists.

### Limitations

Several limitations of the present study should be acknowledged, particularly related to the neglect of potentially relevant variables. First, behavioral measures (e.g., impulsivity) were not assessed, despite their possible influence on cognitive functioning. Additionally, the potential effects of medications acting on the cardiovascular system were not controlled for, which may have affected performance on the HbPT, thereby limiting the interpretability of the findings. A further limitation concerns the clinical characterization of the sample, which was relatively limited and did not include additional pharmacological variables (e.g., medication types and dosages), or motor features (e.g., motor lateralization or motor subtype). These aspects may have hindered a more thorough comparison with existing literature and may have contributed to sample heterogeneity; thus, present findings should be interpreted with caution.

## Conclusions

The present study sought to examine both interoception and MBR, as well as their potential association, in PwPD. Our results extend previous studies, suggesting the possibility of an impairment of both action-oriented and non-action-oriented MBR abilities, also highlighting the importance of preserving both cortical and subcortical cognitive functions for a good MBR ability. In light of these findings and given the importance of these constructs in everyday functioning, the present study suggests (i) to further evaluate interoception in PwPD in order to establish specific interoceptive deficits in PD, (ii) to assess more in detail the body processing in PwPD (i.e., evaluating the performance in all body representations, static and dynamic ones, also exploring it out of the motor imagery field and also using control tasks), (iii) to explore the possible influence of PD staging on both interoception and MBR (i.e., evaluating it through transversal or longitudinal studies). Regarding this latter aspect, it is important to consider that recent literature has highlighted a change of both interoceptive and MBR abilities over time in healthy people (e.g., Murphy et al. 2018; Raimo et al. (Raimo et al. 2021b); so, a better understanding of interoceptive and MBR functioning over time in PwPD is necessary to improve our knowledge of its deviations from typical decline as well as to provide tailored rehabilitation training programs for specific interoceptive and MBR deficits.

## Supplementary Information

Below is the link to the electronic supplementary material.

Supplementary Material 1

Supplementary Material 2

Supplementary Material 3

Supplementary Material 4

## Data Availability

The data presented in this study are available on request from the corresponding author.
