# Supplementary material for: Exploring the relationship between interoception and mental body representation in Parkinson’s disease
Source: J Neural Transm (Vienna). 2026 Jun 6;133(9):2547–56. doi: 10.1007/s00702-026-03194-0 (PMC13687247; doi:10.1007/s00702-026-03194-0)
Supplement: Supplementary file 1 [file 702_2026_3194_MOESM1_ESM.docx]

*Table s2*. Correlational analyses, with Spearman's rho coefficient, between cognitive variables and performance on both interoceptive and body representation tasks in the patients’ group.

|  | MoCA score | PDCRS/AF score |
| --- | --- | --- |
| HbPT- accuracy score | rho=-0.113  p=0.575 | rho=-0.217  p=0.277 |
| HbPT- awareness score | rho=-0.083  p=0.680 | rho=-0.208  p=0.298 |
| SAQ score | rho=-0.133  p=0.527 | rho=0.059  p=0.781 |
| FBE left side score | rho=-0.142  p=0.481 | rho=-0.284  p=0.150 |
| FBE right side score | rho=-0.150  p=0.455 | rho=-0.239  p=0.229 |
| FBE central region score | rho=-0.021  p=0.919 | rho=-0.006  p=0.975 |
| FBE total score | rho=-0.226  p=0.257 | rho=-0.416  p=0.031 |
| HLT left side score | rho=0.374  p=0.055 | rho=0.602  p**<0.001*** |
| HLT right side score | rho=0.373  p=0.055 | rho=0.518  p=**0.006*** |
| HLT total score | rho=0.428  p=0.026 | rho=0.641  p<**0.001*** |

** Significant after Benjamini-Hochberg correction for multiple comparisons.*

*Notes.* MoCA: Montreal Cognitive Assessment; PD-CRS/AF: Parkinson’s Disease - Cognitive Rating Scale/Alternative Form; HbPT: Heratbeat Perception Task; SAQ: Self-Awareness Questionnaire; FBE: Frontal Body Evocation task; HLT: Hand Laterality Task.
