# Supplementary material for: Exploring the relationship between interoception and mental body representation in Parkinson’s disease
Source: J Neural Transm (Vienna). 2026 Jun 6;133(9):2547–56. doi: 10.1007/s00702-026-03194-0 (PMC13687247; doi:10.1007/s00702-026-03194-0)
Supplement: Supplementary file 2 [file 702_2026_3194_MOESM2_ESM.docx]

*Table s3*. Correlational analyses, with Spearman's rho coefficient, between behavioral variables and performance on both interoceptive and body representation tasks in the patients’ group.

|  | BDI-II score | DAS  executive score | DAS  emotional score | DAS  cognitive/  behavioral score |
| --- | --- | --- | --- | --- |
| HbPT- accuracy score | rho=-0.127  p=0.527 | rho=0.145  p=0.472 | rho=-0.356  p=0.068 | rho=-0.099  p=0.623 |
| HbPT- awareness score | rho=-0.077  p=0.701 | rho=-0.287  p=0.147 | rho=0.317  p=0.107 | rho=-0.082  p=0.683 |
| SAQ score | rho=0.148  p=0.481 | rho=0.165  p=0.431 | rho=-0.095  p=0.651 | rho=0.176  p=0.401 |
| FBE left side score | rho=0.197  p=0.324 | rho=0.155  p=0.439 | rho=0.295  p=0.135 | rho=-0.058  p=0.773 |
| FBE right side score | rho=0.175  p=0.383 | rho=0.096  p=0.634 | rho=-0.062  p=0.758 | rho=-0.180  p=0.369 |
| FBE central region score | rho=-0.173  p=0.387 | rho=-0.216  p=0.279 | rho=0.056  p=0.780 | rho=-0.338  p=0.084 |
| FBE total score | rho=0.288  p=0.146 | rho=0.202  p=0.312 | rho=0.125  p=0.535 | rho=-0.024  p=0.905 |
| HLT left side score | rho=-0.036  p=0.858 | rho=-0.057  p=0.779 | rho=0.235  p=0.239 | rho=0.039  p=0.847 |
| HLT right side score | rho=0.003  p=0.988 | rho=0.068  p=0.735 | rho=0.399  p=0.039 | rho=0.304  p=0.123 |
| HLT total score | rho=-0.056  p=0.780 | rho=-0.052  p=0.796 | rho=0.299  p=0.130 | rho=0.121  p=0.549 |

** Significant after Benjamini-Hochberg correction for multiple comparisons.*

*Notes.* BDI-II: Beck Depression Inventory - II; I-DAS: Italian - Dimensionale Apathy Scale; HbPT: Heratbeat Perception Task; SAQ: Self-Awareness Questionnaire; FBE: Frontal Body Evocation task; HLT: Hand Laterality Task.
