# Supplementary material for: Exploring the relationship between interoception and mental body representation in Parkinson’s disease
Source: J Neural Transm (Vienna). 2026 Jun 6;133(9):2547–56. doi: 10.1007/s00702-026-03194-0 (PMC13687247; doi:10.1007/s00702-026-03194-0)
Supplement: Supplementary file 3 [file 702_2026_3194_MOESM3_ESM.docx]

*Table s1*. Correlational analysis, with Spearman's rho coefficient, between clinical variables and performance on both interoceptive and body representation tasks in the patients’ group.

|  | Disease duration | UPDRS-III score | H&Y score |
| --- | --- | --- | --- |
| HbPT- accuracy score | rho=-0.176  p=0.445 | rho=-0.207  p=0.355 | rho=-0.300  p=0.186 |
| HbPT- awareness score | rho=-0.052  p=0.822 | rho=0.133  p=0.555 | rho=0.235  p=0.306 |
| SAQ score | rho=-0.014  p=0.952 | rho=0.156  p=0.499 | rho=-0.013  p=0.957 |
| FBE left side score | rho=0.095  p=0.683 | rho=-0.158  p=0.483 | rho=0.440  p=0.046 |
| FBE right side score | rho=0.112  p=0.630 | rho=0.198  p=0.376 | rho=0.361  p=0.108 |
| FBE central region score | rho=-0.049  p=0.833 | rho=0.614  p=0.002 | rho=0.159  p=0.492 |
| FBE total score | rho=0.140  p=0.544 | rho=-0.051  p=0.820 | rho=0.455  p=0.038 |
| HLT left side score | rho=0.045  p=0.845 | rho=-0.228  p=0.307 | rho=-0.179  p=0.438 |
| HLT right side score | rho=0.113  p=0.625 | rho=-0.053  p=0.815 | rho=-0.022  p=0.924 |
| HLT total score | rho=0.058  p=0.802 | rho=-0.130  p=0.563 | rho=-0.167  p=0.470 |

*Notes.* UPDRS-III: Unified Parkinson's Disease Rating Scale - part III; H&Y: Hoehn and Yahr scale; HbPT: Heartbeat Perception Task; SAQ: Self-Awareness Questionnaire; FBE: Frontal Body Evocation task; HLT: Hand Laterality Task.
