# Supplementary material for: Exploring the relationship between interoception and mental body representation in Parkinson’s disease
Source: J Neural Transm (Vienna). 2026 Jun 6;133(9):2547–56. doi: 10.1007/s00702-026-03194-0 (PMC13687247; doi:10.1007/s00702-026-03194-0)
Supplement: Supplementary file 4 [file 702_2026_3194_MOESM4_ESM.docx]

*Table s4*. Correlational analysis, with Spearman's rho coefficient, between interoception and mental body representation in the patients’ group.

|  | HbPT- accuracy score | HbPT- awareness score | SAQ score |
| --- | --- | --- | --- |
| FBE left side score | rho=0.140  p=0.486 | rho=0.230  p=0.249 | rho=-0.383  p=0.059 |
| FBE right side score | rho=-0.060  p=0.767 | rho=0.166  p=0.407 | rho=-0.045  p=0.829 |
| FBE central region score | rho=-0.168  p=0.401 | rho=-0.034  p=0.867 | rho=-0.209  p=0.317 |
| FBE total score | rho=0.109  p=0.589 | rho=0.223  p=0.264 | rho=-0.286  p=0.166 |
| HLT left side score | rho=-0.090  p=0.656 | rho=-0.180  p=0.370 | rho=0.177  p=0.396 |
| HLT right side score | rho=-0.267  p=0.178 | rho=-0.063  p=0.754 | rho=0.371  p=0.068 |
| HLT total score | rho=-0.136  p=0.499 | rho=-0.145  p=0.469 | rho=0.209  p=0.315 |

*Notes.* HbPT: Heratbeat Perception Task; SAQ: Self-Awareness Questionnaire; FBE: Frontal Body Evocation task; HLT: Hand Laterality Task.
